# Linear and Nonlinear Heart Rate Variability Indexes in Clinical Practice

**DOI:** 10.1155/2012/219080

**Published:** 2012-02-14

**Authors:** Buccelletti Francesco, Bocci Maria Grazia, Gilardi Emanuele, Fiore Valentina, Calcinaro Sara, Fragnoli Chiara, Maviglia Riccardo, Franceschi Francesco

**Affiliations:** Department of Emergency Medicine and Intensive Care, Catholic University of the Sacred Heart, 00168 Rome, Italy

## Abstract

Biological organisms have intrinsic control systems that act in response to internal and external stimuli maintaining homeostasis. Human heart rate is not regular and varies in time and such variability, also known as heart rate variability (HRV), is not random. HRV depends upon organism's physiologic and/or pathologic state. Physicians are always interested in predicting patient's risk of developing major and life-threatening complications. Understanding biological signals behavior helps to characterize patient's state and might represent a step toward a better care. The main advantage of signals such as HRV indexes is that it can be calculated in real time in noninvasive manner, while all current biomarkers used in clinical practice are discrete and imply blood sample analysis. In this paper HRV linear and nonlinear indexes are reviewed and data from real patients are provided to show how these indexes might be used in clinical practice.

## 1. Complexity in Biological Signals

Biological systems are complex systems; particularly, they are systems that are spatially and temporally complex, built from a dynamic web of interconnected feedback loops and marked by interdependence, pleiotropy, and redundancy [1]. The meaning of variability in biological signals was studied by Goldberger [2]. He proposed that increased regularity, of signals represents a “decomplexification” of illness. Thus, health is characterized by “organized variability” and disease is defined by decomplexification, increased regularity and reduction in variability. In contrast to the “decomplexification” hypothesis, Vaillancourt and Newell [3] noted increased complexity and increased approximate entropy in several disease states and hypothesized that disease may manifest with increased or decreased complexity, depending on the underlying dimension of the intrinsic dynamic (e.g., oscillating versus fixed point). In addition to the discussion, Macklem's studies on asthma as a disease of higher energy dissipation, greater distance from thermodynamic equilibrium, lower entropy, and greater variation [4] suggest that health is defined by a certain distance from thermodynamic equilibrium; too close (decreased variation, too little energy dissipation, low entropy) or too far (increased variation and energy dissipation, high entropy) each represents pathological alterations [5].

The host response to sepsis, shock, or trauma is an example of a biological complex system that is readily apparent to intensivists [6]. It is within this complex systems conception of health and illness that the clinical utility of variability analysis may be appreciated and should determine the impact that the variability analysis has on critically ill patient outcome.

If we look at a modern emergency department (ED) and intensive care unit (ICU) we can appreciate a continuous stream of information: parameters derived by multiple monitors and ventilators, laboratory data, and clinical documentation. Usually, data are collecting intermittently but this system is not adequate for tracking and analysis of complex multivariate relationships. Variability analysis represents a novel means to evaluate and treat individual patients, suggesting a shift from epidemiological analytical investigation to continuous individualized complexity analysis [7]. Complexity analysis of time series has been widely used in the study of variability of biological phenomena, as heart rate [8].

Heart rate is probably the easiest biological, complex, signal to analyze. Heart rate, recorded as a space between two heartbeats or as a distance R-R on an surface electrocardiogram (ECG), is irregular if measured in milliseconds. This kind of variation appeared significant and is related to physiological (or pathological) conditions. Previous studies demonstrated a fractal-like complexity pattern in the variability of heart rate (HRV) which is possible to measure and quantify. Rapid fluctuation of HRV can reflect changes of sympathetic and parasympathetic activity; in other words, HRV is a noninvasive index of the autonomic nervous system's control on the heart. Recent studies suggested that mechanisms involved in the regulation of cardiovascular system interact with each other in a nonlinear way and that it is possible to study these mechanisms with several algorithms. Clinically, patients after an acute myocardial infarction showed altered HRV indexes values with such differences correlating to overall mortality [9].

The aim of this paper is to describe different approaches to HRV quantifications in real patients, all of possible utility in future clinical practice.

## 2. Heart Rate Variability Indexes

*See references for a clear and exhaustive explanation of different HRV indexes and their meanings and clinical use* [10].  Table 2 shows most used HRV indexes in clinical practice. 

### 2.1. Linear Algorithms

Using linear algorithms, HRV can be analyzed in time or frequency domain. *Time domain indexes* are the first used indexes and simplest way to calculate HRV, because they are statistical calculations of consecutive RR intervals, and they are strictly correlated with each other (SDNN, SDANN, pNN50, ecc…). *Frequency domain indexes* are more elaborated indexes based on spectral analysis, mostly used to evaluate the contribution on HRV of autonomic nervous system (VLF, LF, HF, HF/LF ratio). Spectral analysis can be used to analyze the sequence of NN intervals of short-term recordings (2 to 5 minutes) or an entire 24-hour period (i.e., Holter-ECG record).

### 2.2. Nonlinear Algorithm

Non linear (fractal) indexes [11] are recently introduced methods to measure HRV, not affected by nonstationarity, as it happens for linear indexes. They include Power Law Exponent, Approximate Entropy and Detrended Fluctuation Analysis. These methods study all complex interactions of hemodynamic, electrophysiological, and humoral variables as well as by the autonomic and central nervous regulations. These techniques have been shown to be powerful tools for characterization of various complex systems, but however no systematic study has been conducted to investigate large patient populations with the use of these methods. 

Starting from frequency analysis, *Power Law Exponent *[12] describes the nature of correlations of single frequencies in a time series. When equal to 1, it states that the time series has similar fluctuations acting at different scales, regardless of the size of the variation (namely. it is “scale invariant,” a property of fractals [13]). It has been applied in biology and medicine formerly to describe the dynamics of beat-to-beat interval in ageing [8]. *Approximate Entropy* (ApEn) [14] provides a measure of the degree of irregularity or randomness within a series of data. Smaller values indicate greater regularity, and greater values convey more randomness and system complexity. It is a rather new index applied in biological systems signals study and it still needs implementation.

### 2.3. Detrended Fluctuation Analysis (DFA)

This method has been developed in order to make a distinction between the internal variations generated by complex systems and those variations caused by some environmental-external stimulus [15]. A singular ECG derivation is recorded continuously and the R-R distance is calculated in milliseconds until it is possible to get an amount of 8000 R-R that are necessary to assure an adequate interval of time. The data's series have been integrated and divided into a series of regular intervals named *n*, included between 1 and 300. For each *n* interval, it has been calculated the “local” fluctuation as the difference compared to a straight line of a linear interpolation. Indeed, the “global” fluctuation has been calculated as the square root of the average of the local's fluctuations.

## 3. Examples of HRV Indexes in Healthy and Critically Ill Patients

HRV indexes were computed using a digital 12 leads ECG-Holter machine (Mortara Instruments, USA) in twenty consecutive patients admitted to the Intensive Care Unit within 24 hours of admission and in an aged-matched (2 : 1 ratio) control population from consecutive patients presenting to the ED with nontraumatic, self-limited, chest pain as chief complain. They were at very low/low/medium risk [16] for Acute Coronary Syndrome (ACS), and entered in a dedicated protocol to be screened for silent cardiac ischemia with serial cardiac enzymes measures (high-sensitivity Troponin T, Elecsys, Roche, Germany) followed by provocative cardiac stress test (either treadmill or nuclear stress test). All patients had to have at least 18 years of age and a baseline 12 leads ECG without diagnostic T-wave or ST-segment deviation suggesting ongoing acute coronary syndrome. Study protocol was approved by Ethical Committee. ECGs were manually reviewed and only patients with sinus rhythm at baseline were eligible for HRV indexes computation. ECG-Holter data were analyzed by Mortara proprietary software to obtain RR intervals in milliseconds, then HRV indexes were manually calculated. Holter data with artifacts or nonsinus beat more than 10% of total beats number were excluded from the analysis.

Critically ill patients consisted in a mixed population treated in the Medical Intensive Care Unit (MICU) and in the Surgical Intensive Care Unit (SICU). The SICU group enrolled patients after major heart surgery (coronary artery bypass graft or valvular replacement) while the MICU patients were treated for septic shock (defined as infection in the setting of high serum lactate and unstable hemodynamic conditions at presentation). All patients were mechanically ventilated and under treatment with Propofol. 

In a study published in 2005 on Anesthesia Analgesia, Propofol induces significant decreases in BP, LF, HF, ApEn, and LF/HF ratio with no change in HR, indicating predominance of parasympathetic activity during sedation. The decreased BP with no change in HR indicates that propofol attenuates the baroreflex reaction [17]. Kanaya et al. [18] reported that continuous infusion of propofol at a rate of 3 mg/kg/min reduced cardiac parasympathetic tone based on a decrease in entropy and HF with no significant changes in LF, LF/HF, and HR. 

**Figure 1:**
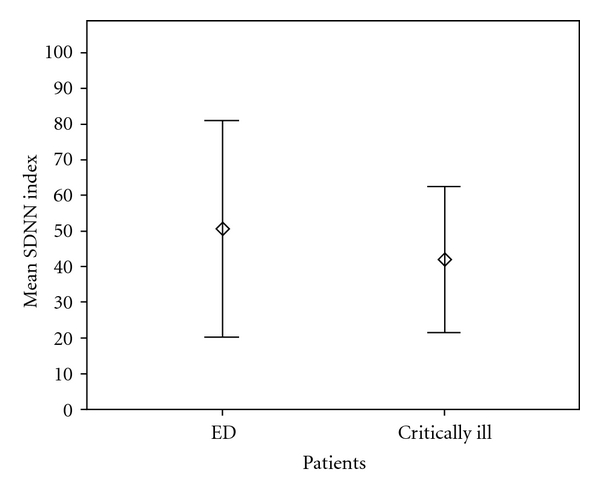
Series Standard Deviation (Frequentist Statistics). SDNN index displayed as mean (circles) and 95% confidential interval (Bars). Healthy subjects showed a higher degree of dispersion around the mean (higher variability) compared to critically ill patients, *P* = 0.10 using Mann-Witney *U*-test. ED: Emergency Department.

**Figure 2:**
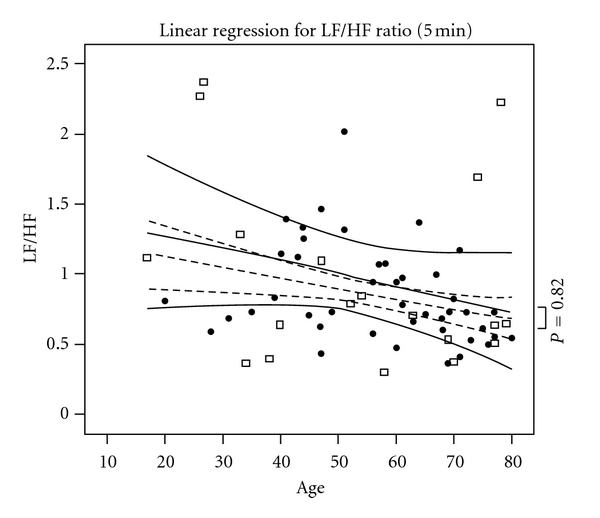
Fast Fourier Transform Analysis. Black dots represent healthy patients and empty squares ICU cases. Dashed and continuous lines reflect LF/HF ratio after adjusting for other clinical comorbidities along with 95% confidential intervals (curved lines). *x*-axis represents age in years. The two groups did not differ in term of LF/HF ratio (*P* = 0.82).

**Figure 3:**
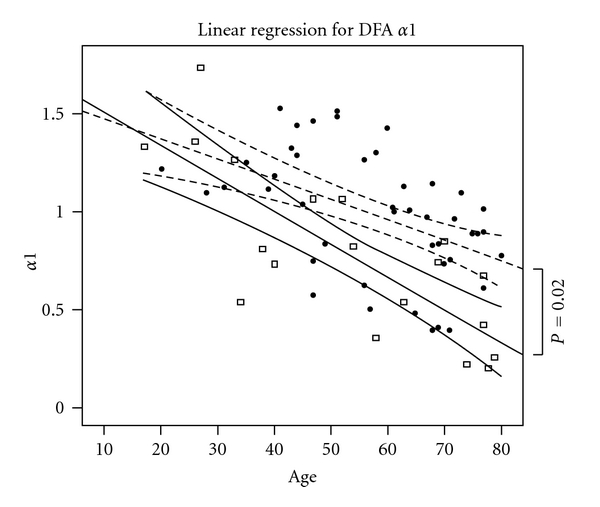
Detrended Fluctuation Analysis (DFA). Black dots represent healthy patients and empty squares ICU cases. Dashed and continuous lines reflect predicted values (adjusted for comorbidities) for the respective group along with 95% confidential intervals (curved lines). *x*-axis represents years. It is to be noted that DFA index was significantly different between the two groups even when adjusted for other comorbidities and age (*P* = 0.02). Age affects DFA index in both groups.

All patients were lying supine and no invasive procedures were performed during ECG signal acquisition. ECG signal was sampled at 1 KHz (1000 samples/sec), assuring a good quality of measurement in a millisecond scale. STATA 11.0 (Stata Corp, TX) was used to compute statistics.  Table 1 provides clinical characteristics of the two groups. Figures 1, 2, and 3 show the three most used HRV indexes behaviours in the two groups.

## 4. Bridging the Gap: From Research to Clinical Practice

As clinicians, ED physicians and intensivists are always interested in predicting patient's risk of developing major and life-threatening complications. Such risk models should include biomarkers of prognostic value. Anticipating clinical course implies a deep knowledge of the present patient's state. Understanding biological signals behavior might represent a fundamental step toward a better care. The main advantage of signals such HRV indexes is that it can be calculated in real time in noninvasive manner. In fact, all current biomarkers used in clinical practice are discrete and imply blood sample analysis. 

Although HRV indexes appear to be appealing, further research is required. First, nonlinear indexes are not standardized in terms of data gathering methodology and minimum numbers of R-R intervals needed to have back a reliable measure. Second, HRV indexes represent the final outcome of complex systems. For instance, it is known that diabetes significantly affects final results, along with aging. All those chronic clinical characteristics are not well studied and no nomograms exist to simply adjust indexes results for these covariates. Third, it is not clear how and why different algorithms behave in different manner. For instance, fast Fourier analysis was shown to provide information and was able to discriminate between patients with and without coronary artery disease, but it seemed not to be the same case in our example (although sample size is similar in our case and in previous study [19]).

## 5. Conclusions

Different pathophysiologic processes alter HRV indexes in opposite directions, making it difficult to identify them when present at the same time. For instance, age and diabetes both decrease DFA index while acute myocardial ischemia seems to increase it. HRV-indexes future studies should be aimed to evaluate how HRV is affected by known cardiovascular risk factors and to find a “standard” of measurement of different indexes, comparing healthy and ill patients and investigating their risk of major cardiovascular events.

In conclusion, future larger studies are warranted before HRV indexes can be embedded into daily clinical practice as routine standard of care.

## Figures and Tables

**Table 1:** Descriptives. All variables are displayed as mean (standard deviation) except gender expressed as number (%).

	Critically Ill Patients (*n* = 20)	ED Patients (*n* = 45)
Age (years)	54 (20)	57 (14)
Gender (Male)	15 (75)	25 (55)
SAPS	38 (17)	—
SOFA	6.3 (4.6)	—
APACHE II	15 (7)	—
SBP (mmHg)	132 (23)	140 (23)
DBP (mmHg)	73 (24)	80 (20)
Heart Rate (bpm)	81 (19)	80 (19)
SDNN index (ms)	40 (21)	53 (34)
LF/HF Ratio 5 min (ms^2^)	0.97 (0.67)	0.84 (0.34)
LF/HF Ratio tot. (ms^2^)	0.89 (0.40)	0.85 (0.23)
DFA		
Alpha1	0.76 (0.43)	0.98 (0.31)
Alpha2	0.99 (0.18)	1.01 (0.09)

ED: emergency Department. SBP: systolic blood pressure. DBP: diastolic blood pressure. SDNN: standard deviation of the NN intervals. LF: low frequency. HF: high frequency. DFA: detrended fluctuation analysis.

**Table 2:** Most used HRV measures. (modified from *Heart Rate Variability: Standards of Measurement, Physiological Interpretation, and Clinical Use. Task Force of the European Society of Cardiology the North American Society of Pacing Electrophysiology. Circulation. 1996;93:1043*–*1065*).

Time domain indexes	SDNN	Standard deviation of all NN intervals
	SDANN	Standard deviation of the averages of NN intervals in all 5-minute segments of the entire recording
	RMSSD	The square root of the mean of the sum of the squares of differences between adjacent NN intervals
	SDNN index	Mean of the standard deviations of all NN intervals for all 5-minute segments of the entire recording
	pNN50	NN50 count divided by the total number of all NN intervals

Frequecy domain indexes	Total power	Variance of all NN intervals (≤0.4 Hz)
	ULF	Power in the ULF range (≤0.003 Hz)
	VLF	Power in the VLF range (0.003–0.04 Hz)
	LF	Power in the LF range (0.04–0.15 Hz)
	HF	Power in the HF range (0.15–0.4 Hz)
	LF/HF	Ratio LF [ms^2^]/HF[ms^2^]
